# The microRNA-10b-Bim axis promotes cancer progression through activating autophagy in oral squamous cell carcinoma

**DOI:** 10.1038/s41420-022-01168-1

**Published:** 2022-08-25

**Authors:** Shaoming Li, Ling Gao, Jiacheng Liu, Chao Guo, Jingjing Zheng, Keqian Zhi, Wenhao Ren

**Affiliations:** 1 Department of Oral and Maxillofacial Surgery, the Affiliated Hospital of Qingdao University, No.1677 Wutaishan Road, Qingdao, 266555 China; 2 School of Stomatology of Qingdao University, Qingdao, 266003 China; 3 Key Lab of Oral Clinical Medicine, the Affiliated Hospital of Qingdao University, Qingdao, 266555 China; 4 Department of Endodontics, the Affiliated Hospital of Qingdao University, Qingdao, 266555 China

**Keywords:** Tumour biomarkers, Oral cancer

## Abstract

Autophagy is related to many cellular mechanisms and dysregulation of autophagy involves the pathological process in cancer. miR-10b activates autophagy, which promotes invasion and migration of OSCC. Its functional role in the mechanism of OSCC to autophagy remains to be unclear. Overexpression of miR-10b was followed by enhanced OSCC invasion and migration and activated autophagic protein, such as LC3II/ATG5. MiR-10b attracted Bim directly according to the Bio-informatics analyses and double luciferases reporter assays. Functional experiments further revealed that miR-10b could promote invasion and migration in vitro. In addition, miR-10b induced autophagy via inhibiting Bim in invasion and migration of OSCC. Notably, animal experiments confirmed that miR-10b-Bim promoted proliferation and autophagy in OSCC. In addition, this study provides a theoretical support for regulating the mechanism of OSCC by inducing autophagy with miR-10b-Bim as a target.

## Introduction

Oral cancers are the most common malignancies encountered in the mouth and maxillofacial region, and more than 90% of them are squamous cell carcinomas [1, 2]. Although the diagnosis and treatment technique of OSCC has improved, the 5-year survival rate still low [3]. As a malignant tumor, invasion and migration were associated with tumor metabolism in malignant progression of OSCC. The invasion and migration of tumors may be influenced by autophagy due to the discovery of more molecular mechanisms [4]. Thus, it is imperative that we explore the molecular mechanism of OSCC and identify new therapeutic targets.

Tumors, malignant transformations, neurological diseases, and inflammatory diseases are thought to be caused by autophagy. An autophagic process may have both positive and negative effects [5]. In the early stage of cancer, autophagy is over-induced, which can cause cell death and exert a tumor suppressor effect; in the advanced stage of cancer, cells maintain autophagy activity to meet their own energy needs and promote cell survival. Both ATG5 and LC3II has been identified as an autophagy substrate and promoted tumor progression under diverse autophagy conditions. Therefore, autophagy protein may serve as a marker for diagnostic and clinicopathological characteristics. Understanding the mechanism of autophagy will provide new diagnosis and treatment strategies for OSCC.

MicroRNA (miRNA) is a type of non-coding RNA composed of about 22 nucleotides. miRNAs reduce the expression level of target genes by degrading mRNA or inhibiting the translation of mRNA, and regulate a variety of cell activities (cell growth, differentiation, metabolism, death, etc.) [6, 7], Therefore, miRNA could participate in various types of regulation in tumor. At present, a variety of miRNA-based therapies have entered the clinical research stage, and miRNA-targeted therapy is expected to become an important tumor treatment method in the future [8]. In addition, miRNAs could regulate autophagy-related genes and further participate autophagy in the tumors [9], which participating in various types of regulation in tumor [10]. We found differences in miR-10b and miR-21 expression between tumor and normal tissues through GEO database analysis [9]. We found statistically differences between OSCC and normal tissue samples when it came to the regulation of cell proliferation, apoptosis, migration, invasion and autophagy by miR-21 [9, 11, 12]. However, whether miR-10b could regulate autophagy in OSCC cells is unclear.

The purpose of this study was to explore the mechanism of regulation of miR-10b in OSCC by collecting 20 pairs of clinical samples and finding that miR-10b expression increased dramatically in OSCC tissues. Subsequent studies found that miR-10b can promote OSCC cell proliferation, migration, and invasion, and inhibit OSCC cell apoptosis by regulating autophagy. In addition, we screened out the target gene BCL2L11(Bim) of miR-10b in OSCC by bioinformatics analysis, and firstly verified the regulatory relationship between miR-10b and Bim, revealing the regulatory mechanism of miR-10b on OSCC cell autophagy.

## Results

### miR-10b is upregulated in OSCC specimens and cell lines

**Fig. 1:**
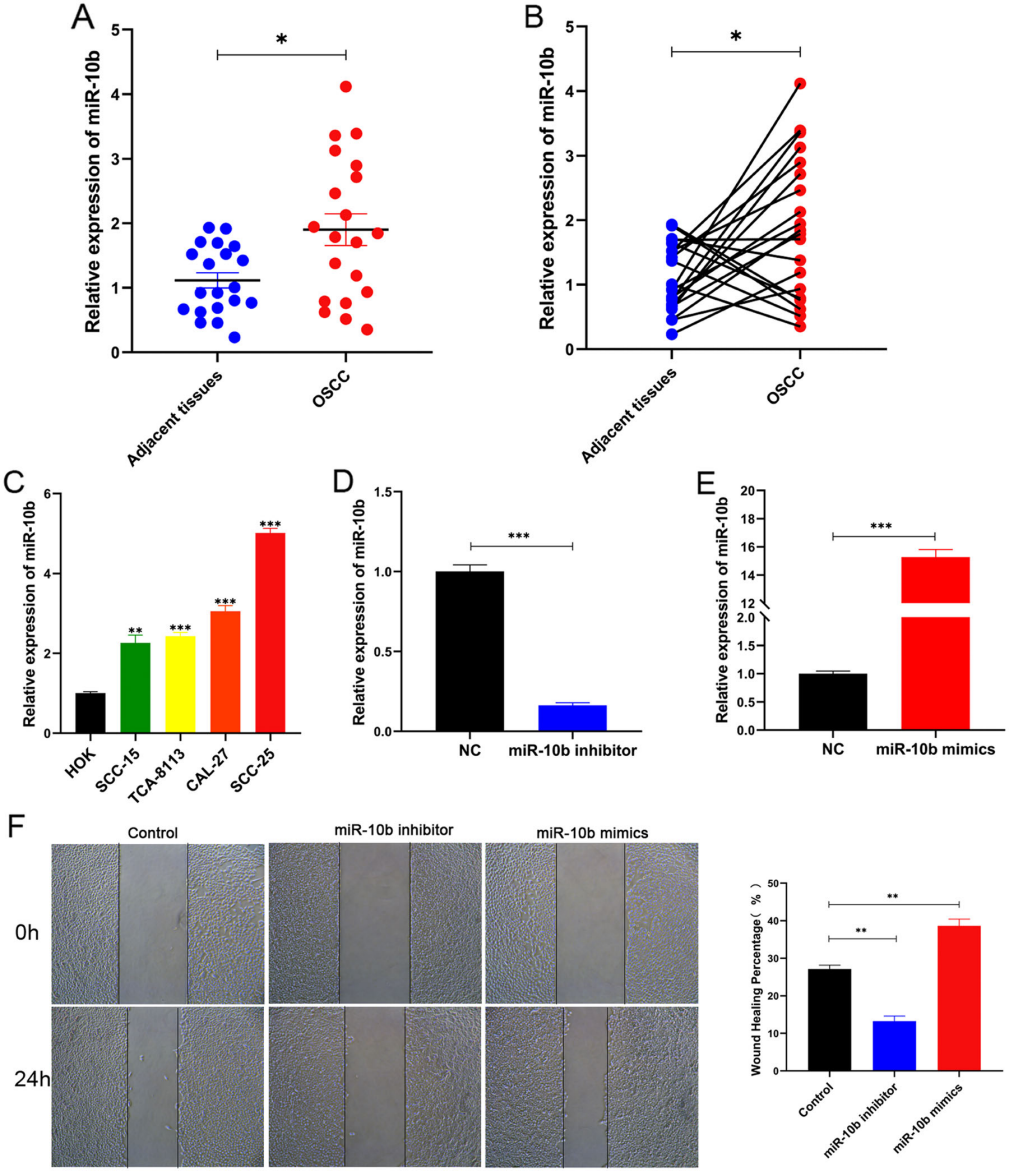

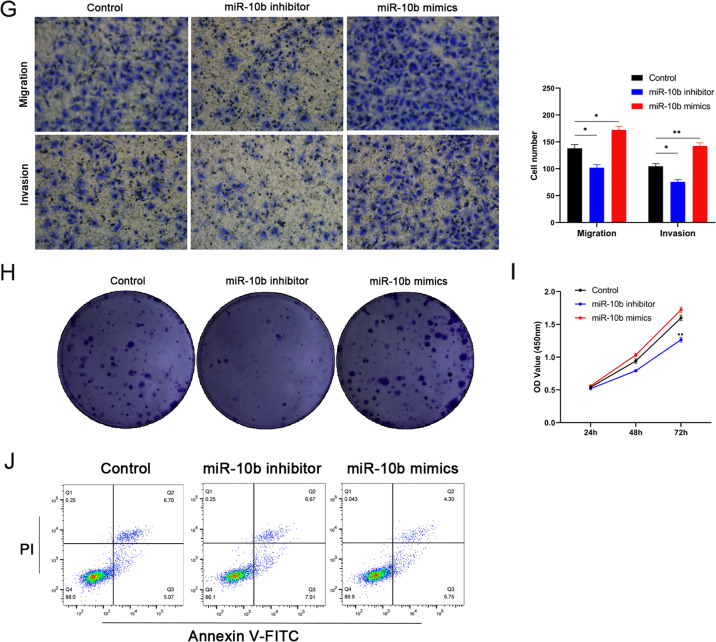
Confirmation of expression of miR-10b in OSCC specimens and cell lines. **A**, **B**, miR-10b expression in 20 pairs of OSCC specimens and paired adjacent tissues was detected by RT-qPCR. **C** The expression of miR-10b in cell lines was examined using RT-qPCR. **D**, **E** miR-10b expression was detected in SCC-25 cells transfected with miR-10b inhibitor or mimics. **F** Wound-healing assay transfected with miR-10b inhibitor or mimics for SCC-25 cells. **G** Migration and invasion assays transfected with miR-10b inhibitor or mimicsfor SCC-25 cells by transwell. **H** In SCC-25 cells transfected with miR-10b inhibitors or mimics, colony formation assays were performed. **I** Proliferation capacities of SCC-25 cells transfected with miR-10b inhibitor or mimics were assayed at 24, 48, and 72 h by CCK-8. **J** Flow cytometry was used to detect cell apoptosis in SCC-25 cells transfected with miR-10b inhibitors and mimics. *p < 0.05, **p < 0.01.

We performed a miRNA microarray in 20 pairs of OSCC tissues and paired adjacent normal tissues. The expression level of miR-10b was significantly higher in OSCC than in adjacent tissues based on real-time RT-qPCR (Fig. 1A, B, *p* < 0.05). To explore the functional role of miR-10b in OSCC, we first detected the expression of miR-10b in OSCC cell lines and human oral keratinocyte cells (HOK). MiR-10b was highly expressed in the four cell lines (Fig. 1C, *p* < 0.01, *p* < 0.001). We found that miR-10b expression was more predominant in OSCC tissues compared to that in adjacent normal tissues. MiR-10b mimics, inhibitors, or the equivalent negative control were transfected into OSCC cells to study its effects on OSCC mechanism (NC.10b). RT-qPCR revealed successful establishment of miR-10b overexpression and suppression cell model (Fig. 1D, E, *p* < 0.001). The number of migrating cells transfected with miR-10b mimics were increased significantly (Fig. 1F, *p* < 0.01), compared with miR-10b inhibitors group. Migrating and invasive cells were significantly increased by miR-10b mimic transfection in transwell chambers (Fig. 1G, *p* < 0.01). In SCC-25 cells, the number of colonies decreased when miR-10b inhibitors were transfected, and proliferation viability was enhanced when miR-10b mimics were transfected (Fig. 1H, I, *p* < 0.05). Moreover, SCC-25 cells with transfected miR-10b inhibitors was increased the apoptosis rates (Fig. 1J, *p* < 0.05). Based on these findings, miR-10b regulates OSCC cell migration, invasion, and apoptosis.

### miR-10b are correlated with autophagy in OSCC cells

**Fig. 2:**
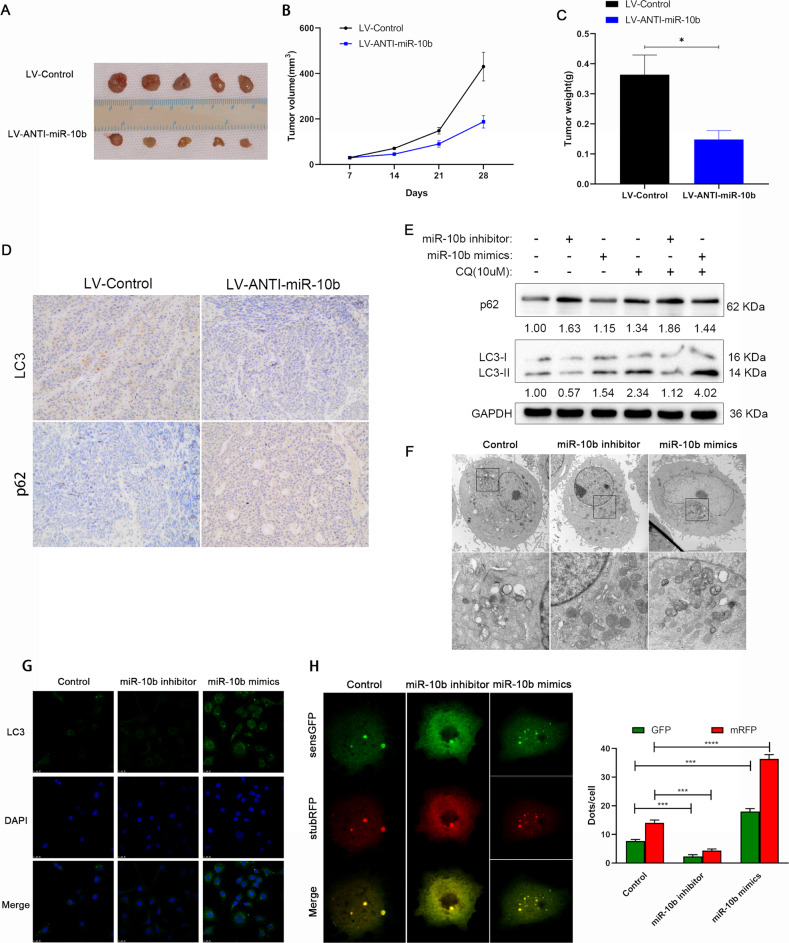
miR-10b are correlated with autophagy in OSCC cells. **A** Tumor growth 28 days after OSCC cells were implanted subcutaneously. **B**, **C** Volume and weight of tumors in LV-ANTI-miR-10b and LV-control groups were calculated. **D** The expression of LC3 and p62 was measured by immunohistochemistry staining (scale bar, 200 μm). *p < 0.05. **E** Western blot analysis was used to detect LC3-II and P62 expression in SCC-25 cells treated with negative control, Mir-10b inhibitor, and Mir-10b mimics (10 μM CQ for 2 h). **F** Transmission electron microscopy (TEM) was used to detect the autophagic microstructure of SCC-25 cells (scale bar, 500 nm). **G**, **H** Volume and weight of tumors in LV-ANTI-miR-10b and LV-control groups were calculated. *p < 0.05, **p < 0.01, ***p < 0.001.

Autophagy plays a double-edged sword role in tumors [13], LC3 and SQSTM1 (p62) are often used to monitor changes in autophagy by Western Blot [14]. To verify whether miR-10b were correlated with autophagy in OSCC cells, An ANTI-miR-10b lentivirus (LV-ANTI-miR-10b) or a control lentivirus (LV-control) was subcutaneously injected into ten nude mice, including LV-ANTI-miR-10b group and LV-control group. It took 7 days for tumors to form after injection, and they were collected on day 28 (Fig. 2A, *p* < 0.05). Tumors grown on SCC-25 cell tumor xenografts in nude mice with overexpression of LV-ANTI-miR-10b were reduced in volume and weight (Fig. 2B, C, *p* < 0.05). These data suggested that tumor growth was significantly accelerated by overexpression of miR-10b in comparison to controls. IHC showed that LC3 was decreased and p62 was increased in the LV-ANTI-miR-10b group (Fig. 2D, *p* < 0.05). We found that LC3-II expression was decreased and p62 expression was increased significantly in miR-10b inhibitor group (Fig. 2E). By transmission electron microscope (TEM), it was found that low levels of miR-10b inhibit autophagy, whereas high levels of miR-10b promote autophagy (Fig. 2F). The number of autophagosomes and autophagolysosomes will vary with miR-10b expression (Fig. 2G). Accumulation of sensGFP-stubRFP-LC3 pots were decreased after miR-10b inhibition (Fig. 2H). Based on these data, miR-10b may play an essential role in regulating autophagy, which may partially explain miR-10b-mediated OSCC progression and development.

### Autophagy inhibition reverse the carcinogenic effects of miR-10b in OSCC

**Fig. 3:**
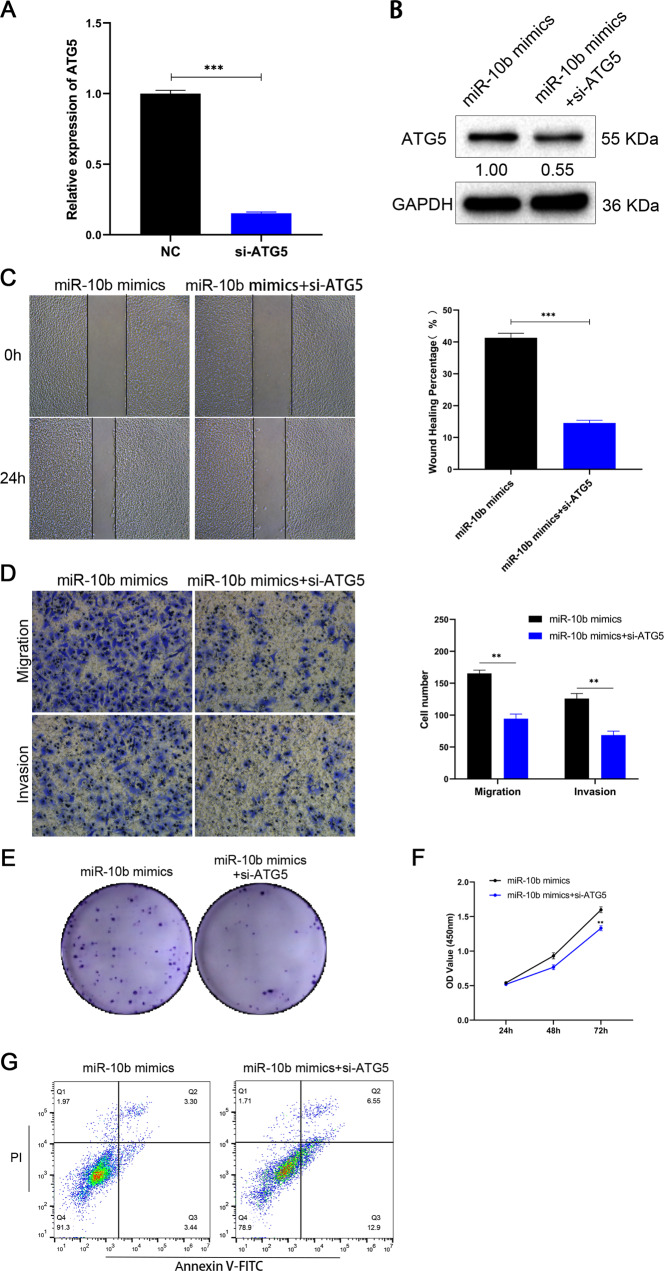
The relationship between autophagy inhibition and miR-10b effects in OSCC cells. **A** miR-10b mimics used to transfected SCC-25 cells with or without si-ATG5, and expression of miR-10b was evaluated using RT-qPCR. **B** The expression of ATG5 protein in SCC-25 cells was detected with western blot. **C** Wound-healing assay for SCC-25 cells transfected with miR-10b mimics or si-ATG5. **D** Migration and invasion assays for SCC-25 cells transfected with miR-10b mimics or si-ATG5 by transwell. **E** Colony formation assays were conducted on SCC-25 cells transfected with miR-10b mimics or si-ATG5. **F** Proliferation capacities of SCC-25 cells transfected with miR-10b mimics or si-ATG5 were assayed at 24, 48, and 72 h by CCK-8. **G** Flow cytometry was used to detect apoptosis in SCC-25 cells transfected with miR-10b inhibitors or si-ATG5. **p < 0.01, ***p < 0.001.

Analyzing the relationship between autophagy and miR-10b’s carcinogenic effects in OSCC cell migration and invasion, this study used si-RNA to inhibit the key autophagy protein ATG5, and explored the transfection efficiency of si-ATG5 was verified by RT-qPCR and Western Blot (Fig. 3A, B, *p* < 0.001). In SCC-25 cells, miR-10b mimics and miR-10b mimics with si-ATG5 were transfected. The scratch experiment showed that the si-ATG5 transfected cells had wider scratches than the control cells after 24 h, and their migration ability was weakened after autophagy inhibition (Fig. 3C, *p* < 0.01). The transwell experiment showed that SCC-25 migrating cells and invaded cells transfected with si-ATG5 and miR-10b mimics was significantly decreased (Fig. 3D, *p* < 0.01). In SCC-25 cells, si-ATG5 transfection significantly reduced the number of colonies (Fig. 3E, *p* < 0.01). Moreover, we found the same result in CCK-8 assay, the downregulation of si-ATG5 could decrease the cell viability (Fig. 3F, *p* < 0.01). Our findings further explored the role of miR-10b in OSCC by showing that si-ATG5 downregulation increased apoptosis (Fig. 3G). These findings indicated that inhibition of ATG5 could reverse the tumor-promoting effect of miR-10b in OSCC cells.

### Bim is a functional target of miR-10b in OSCC

**Fig. 4:**
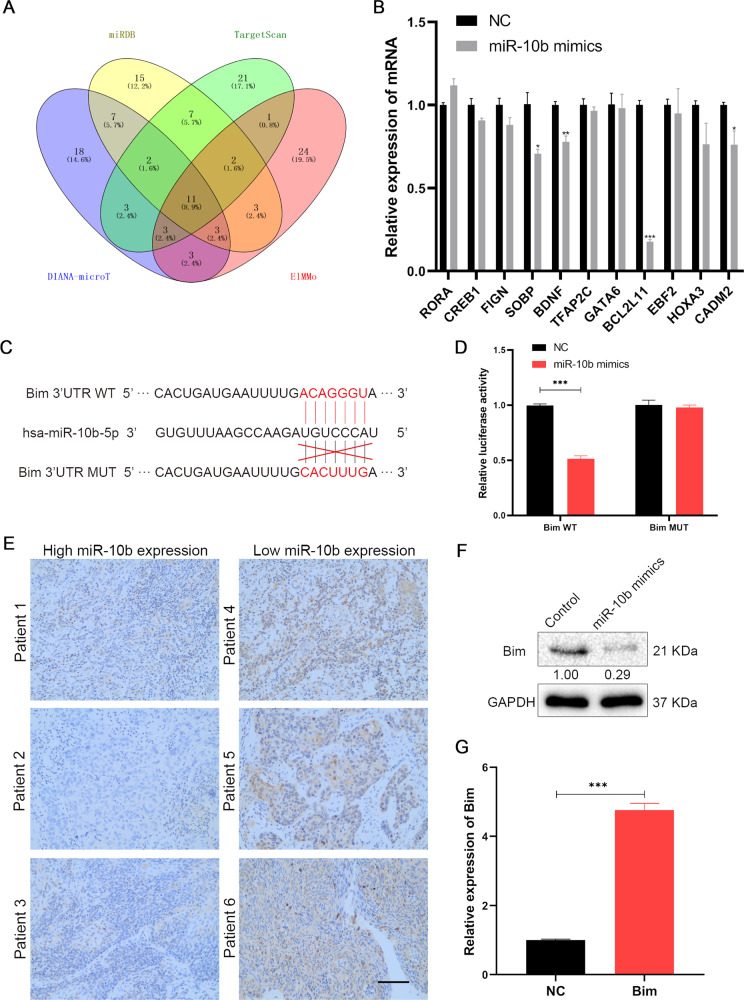
Associations between the Bim and miR-10b in OSCC. **A** Venn diagram of the overlapping parts of the four sets of databases. **B** Eleven candidate genes in total were common to all databases sets, and the expression of candidate genes was detected by RT-qPCR. **C** The miR-10b binding site on Bim predicted by TargetScan. **D** Luciferase reporter assays verify the association between miR-10b and Bim. **E** Immunohistochemical staining of Bim in OSCC tissues. **F** The expression of Bim in protein level detected with western blot. **G** Bim plasmid used to transfected SCC-25 cells, and expression of Bim was evaluated using RT-qPCR. *p < 0.05, **p < 0.05, ***p < 0.05.

In order to explore target genes regulated by miR-10b in OSCC, we used four databases including TargetScan, miRDB, microTCDS, and EIMMo to screen target gene bioinformatics. We obtained the top 50 genes from the four prediction databases (Fig. 4A). 11 candidate genes (RORA, CREB1, FIGN, SOBP, BDNF, TFAP2C, GATA6, BCL2L11, EBF2, HOXA3, and CADM2) were finally determined. Subsequently, SCC-25 cells transfected miR-10b mimics and control cells, RT-qPCR showed that Bim was decreased significantly as potential target gene of miR-10b from 11 candidate genes in OSCC (Fig. 4B, *p* < 0.001). Bim dual luciferase wild-type (WT) and mutant (MUT) 3′-UTR plasmids were constructed based on the potential binding site sequence of miR-10b and the target gene (Fig. 4C). Bim 3′-UTR WT and miR-10b mimics co-transfected into SCC-25 cells significantly decreased luciferase activity compared with the control group (Fig. 4D, *p* < 0.001). Immunohistochemical staining showed that miR-10b regulated the Bim reporter gene from 6 tissue samples (Fig. 4E). Furthermore, OSCC cells treated with miR-10b mimics expressed less Bim protein (Fig. 4F). Bim was confirmed to be the target of miR-10b in OSCC cells based on these data. In order to further analyze the role of Bim in OSCC, this study constructed a overexpression of Bim plasmid and successfully overexpressed Bim mRNA in SCC-25 cells (Fig. 4G).

### miR-10b promotes proliferation, migration, invasion and inhibits apoptosis via regulating Bim expression in OSCC cells

**Fig. 5:**
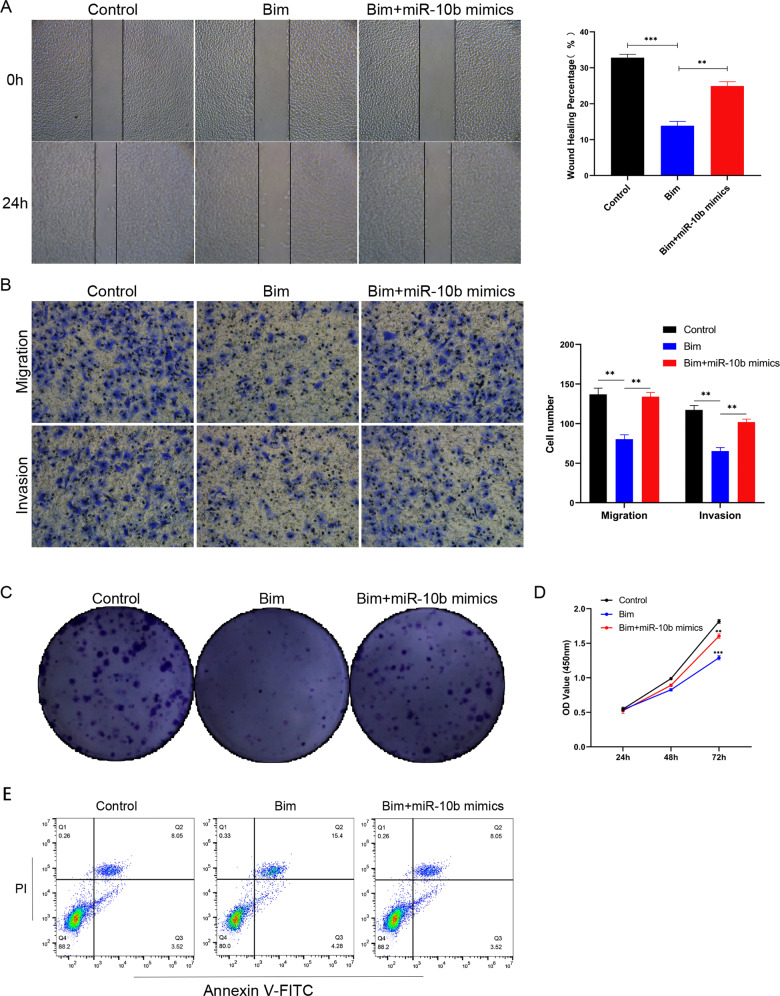
Correlation between miR-10b and Bim in invasion, migration, and apoptosis of OSCC cells. **A** Wound-healing assay for SCC-25 cells transfected with Bim or Bim with miR-10b mimics. **B** Migration and invasion assays for SCC-25 cells transfected with Bim or Bim with miR-10b mimics by transwell. **C** Colony formation assays were performed in SCC-25 cells transfected with Bim or Bim with miR-10b mimics. **D** Proliferation capacities of SCC-25 cells transfected with Bim or Bim with miR-10b mimics were assayed at 24, 48, and 72 h by CCK-8. **E** Cell apoptosis was detected by flow cytometry in SCC-25 cells transfected with Bim or Bim with miR-10b mimics. *p < 0.01, **p < 0.001.

As a member of the BCL2 family, Bim plays a tumor suppressor effect in a variety of cancers [15, 16]. Overexpression of Bim inhibited SCC-25 cells’ migration ability in wound-healing assays (Fig. 5A, *p* < 0.001). When Bim and miR-10b mimics were co-transfected, Bim had an effect on the migration ability of SCC-25 cells. A significant reduction in migration and invasion cells may occur when Bim is overexpressed (Fig. 5B, *p* < 0.01). Similarly, after co-transfection with miR-10b, the inhibition of SCC-25 by Bim was reversed (Fig. 5B, *p* < 0.01). The number of colonies in SCC-25 cells was evidently decreased by overexpression of Bim (Fig. 5C). Moreover, we found the same result in CCK-8 assay. The overexpression of Bim could decrease the cell viability (Fig. 5D, *p* < 0.001). In order to gain a deeper understanding of Bim’s role in OSCC, we found overexpression of Bim increased the apoptosis rates (Fig. 5E). It was found that Bim could effectively inhibit the migration and invasion of OSCC cells. And miR-10b, as its upstream regulatory factor, could effectively reverse the tumor suppressor effect of Bim on OSCC cells. Based on these findings, Bim played a role in cell invasion and migration, and miR-10b targeted it.

### miR-10b-Bim axis is associated with invasion and migration by medicating autophagy in OSCC

**Fig. 6:**
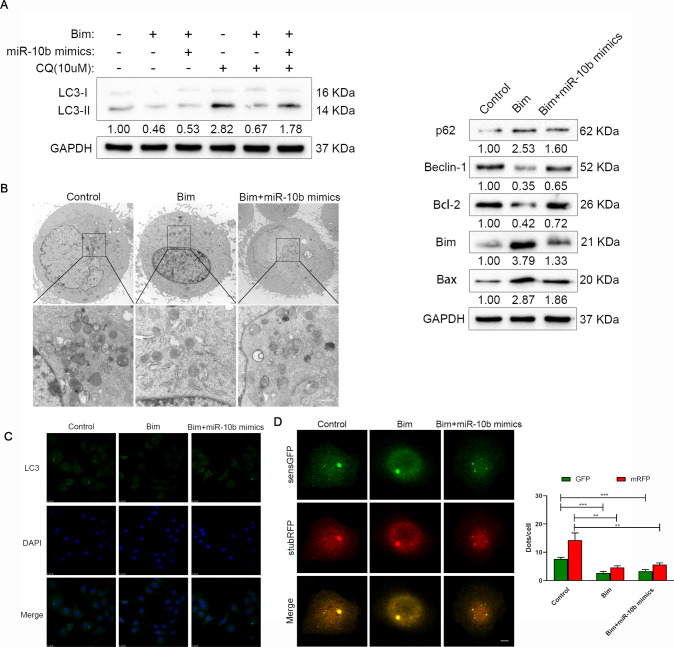
OSCC cells autophagy mediated by the miR-10b-Bim axis. **A** SCC-25 cells were cultured with or without chloroquine (CQ, 10 μM for 2 h). The expression levels of LC3, p62, Beclin-1, Bcl-2, Bax, and Bim were tested by western blotting. **B** Transmission electron microscopy (TEM) was used to detect the autophagic microstructure of SCC-25 cells (scale bar, 500 nm). **C** Immunofluorescent staining showing the number of LC3 dot (green) in the SCC-25 cells. **D** The yellow and red puncta in SCC-25 cells infected with stubRFP-sensGFP-LC3 Adenovirus were observed in confocal culture dishes (scale bar, 10 μm).

As a BCL-2 family protein containing only the BH3 domain, Bim is involved in the autophagy process of OSCC cells [17]. This experiment further explored the role of Bim in OSCC autophagy. The BCL-2 family participated in the regulation of autophagy through its unique BH domain Beclin-1 was an important autophagy-related gene [18], which interacted with the BCL-2 family through its BH3 domain [17]. Bim plasmid could promote Bim protein expression, while miR-10b mimics reversed Bim protein expression. It has been found that Bim significantly inhibits LC3-II and increases p62 expression, and it would reduce the change of autophagy flux after CQ treatment (Fig. 6A), suggesting that Bim could inhibit autophagy in OSCC cells. When Bim was overexpressed, Beclin-1 protein expression was significantly reduced (Fig. 6A), suggesting that Bim inhibited autophagy through the Beclin-1 pathway. When miR-10b was co-transfected, the inhibitory effect of Bim on autophagy was partially reversed (Fig. 6A). Moreover, Bim could suppression Bcl-2 expression and increased the Bax expression (Fig. 6A). To further investigate miR-10b-Bim’s role in autophagy, overexpression of Bim was observed to significantly inhibit autophagosomes and autophagolysosomes by TEM, whereas miR-10b reversed this effect (Fig. 6B). In addition, immunofluorescent staining confirmed that Bim overexpression effectively decreased the expression levels of LC3 (Fig. 6C). The accumulation of sensGFP-stubRFP-LC3 yellow spots decreased after Bim expression, but was partially reversed after miR-10b overexpression, which was consistent with the previous results (Fig. 6D). All data suggest that miR-10b-Bim axis regulated autophagy in OSCC cells.

## Discussion

There is increasing evidence pointing that autophagy played a role in various tumors and other cancer metastases [19]. Some micro-RNAs were associated with autophagy in tumor progression. Therefore, understanding of the molecular mechanisms between miRNA and autophagy was crucial for effective diagnosis and management in OSCC [20, 21].

We collected 20 tissues from OSCC patients and adjacent tissues for this study, and we investigated that expression of miR-10b and miR-21 was increasing in OSCC through gene chip-miRNAs and bioinformatics data. RT-qPCR showed that OSCC cells expressed miR-10b at a statistically different level. For the first time, we found that OSCC is also regulated by miR-10b. As compared to control groups, transfected miR-10b mimics enhanced invasion and migration in cell cultures. It is found that reducing the expression of miR-10b will significantly weaken the migration and invasion ability of OSCC cells, and up-regulating the expression of miR-10b will enhance the migration and invasion ability of OSCC cells. MiR-10b is involved in regulating the migration and invasion ability of OSCC cells, and inhibiting miR-10b can effectively inhibit the carcinogenic effect of miR-10b [22]. The expression of miR-10b increased the size and volume of OSCC tumors in nude mice, implying that miR-10b overexpression significantly accelerated OSCC tumor growth. As a result of these findings, miR-10b plays a critical role in regulating invasion and migration, which might partially explain how miR-10b influences OSCC development and progression in vivo and vitro. In order to verify the specific mechanism by which miR-10b affects OSCC, we conducted the corresponding experiments on cells and tissues.

Our cells experiments indicated that expression of autophagy protein ATG5 and LC3-II was increased in OSCC cells, which might suggest autophagy was associated with invasion and migration of OSCC. We proved for the first time that the up-regulation of miR-10b changed the expression levels of LC3-II and p62 in OSCC cells, and increased autophagy and autophagy flux in OSCC cells [23]. Conversely, the down-regulation of miR-10b inhibited autophagy in OSCC cells. It is suggested that miR-10b is involved in the regulation of autophagy in OSCC cells, and subsequent experiments further confirmed this conclusion (Fig. 2F, H).

We found downstream genes of miR-10b via bio-information and cell experiments. miR-10b targeted down-stream genes, such as Bim, TFAP2C, and GATA6. Cell experiments found that expression of Bim was statistically different in OSCC cells. BIM is an important anti-cancer and pro-apoptotic member of the Bcl-2 family. In autophagy and apoptosis, molecular regulatory mechanisms are primarily controlled by BIM [24]. Based on bio-informatics analysis, miRNA-10b binds to the 3′-UTR of Bim mRNA, and luciferase reporter assay confirms that miRNA-10b can directly act on the 3′-UTR of Bim mRNA, and it was shown that miRNA-10b inhibited the expression of Bim protein by WB. Hence, our data demonstrated that Bim was directly downstream target of miR-10b, and Bim could effectively inhibit the migration and invasion of OSCC cells.

Our study further assessed the potential signaling pathway of the Bim by mediated miR-10b on autophagy. Based on immunofluorescent staining, this study confirms that miR-10b suppresses Bim in OSCC cells, resulting in significantly higher levels of autophagy than controls (Fig. 6C). Studies have found that up-regulation of Bim can effectively inhibit the expression of LC3-II and enhance the accumulation of p62, and inhibit autophagy in OSCC cells. We also observed that Beclin-1 protein expression decreased with the increase in Bim, indicating that the Bim/Beclin-1 pathway is involved in OSCC cell autophagy (Fig. 6A). TEM observed that miR-10b overexpression could significantly increase the number of autophagosomes and autophagolysosomes by suppressing Bim (Fig. 6B). The rescue experiments further demonstrated that miR-10b affects the autophagy of OSCC cells by suppressing Bim (Fig. 6C). Furthermore, we found that Beclin-1 protein expression decreased with increasing Bim, and miR-10b was associated with the Bim/Beclin-1 pathway in OSCC cells. These data firstly showed that miR-10b could inhibit levels of Bim, and miR-10b-Bim could regulate autophagy in invasion and migration of OSCC.

To the best of our knowledge, our results demonstrated that miR-10b induced autophagy in OSCC cells, promoting invasion and migration. Autophagy may be contributing to miR-10b expression and the down-regulation of OSCC Bim. In summary, a new biomarker, miR-10b-Bim axis, has been identified as a potential target in the treatment of OSCC. Therefore, miR-10b-Bim may be useful in diagnosing and treating cancer.

## Materials and methods

### Patients and specimens

Specimens were collected from 20 patients undergoing surgical treatment in the Department of Oral and Maxillofacial Surgery, the Affiliated Hospital of Qingdao University. Patient consent was obtained before obtaining samples, which were collected during surgery in accordance with a hospital’s ethics committee protocol.

### Cell culture and transfection

TCA-8113, SCC-15, SCC-25, CAL-27, and HOK cell lines were all from the cell bank in our research group. The OSCC cell lines culture procedure employed was described in our previous research [25]. The miR-10b inhibitor and miR-10b mimics were used to transfect SCC-25 cells, and constructed by GenePharma (Shanghai, China). Using Lipofectamine3000 (Thermo Fisher Scientific, USA), all transfections were conducted according to the manufacturer’s protocol.

### Quantitative reverse transcription PCR

The total RNA was isolated by using Trizol reagent (Invitrogen, USA) from OSCC cell lines or tissues. cDNA was synthesized from RNA using A Prime Script RT reagent kit (Takara, Japan). A CFX96 Real-Time PCR Detection System (Bio-Rad, USA) and SYBR Green PCR Master Mix (Takara, Japan) were used to perform quantitative RT-PCR. Relative expression of mRNA was determined by the method of 2^−ΔΔCt^ and was normalized to GAPDH. Primer sequences could be found in Table 1.

**Table1:** The sequence of primers.

Gene	Sequence	
miR-10b	RT: GTCGTATCCAGTGCGTGTCGTGGAGTCGGCAATTGCACTGGATACGACcacaaat	
	F: TGCTTACCCTGTAGAACCGA	R: CCAGTGCAGGGTCCGAGGT
U6	F: CGCTTCGGCAGCACATATACTA	R: GGAACGCTTCACGAATTTGC
ATG5	F: AGATGTGTGGTTTGGACGAATTC	R: GAAATCCATTTTCTTCTGCAGGAT
GAPDH	F: CATGTTCGTCATGGGTGTGAA	R: GGCATGGACTGTGGTCATGAG
RORA	F: ACCTACTCCTGTCCTCGTCAGAA	R: GCTGTCTCTCTGCTTTTTTGACATT
CREB1	F: CCAAAAGCGAAGGGAAATTCT	R: GAGAGTCACATGAGCGGCTTT
FIGN	F: ATGTACAGAATGCCCGACAACA	R: CCTACTGGACTGGCTGCTGAA
SOBP	F: GCAGTGTGCCCATTATTGTACCT	R: CTTTTCACCTCACTTCCCATACTCA
BDNF	F: AAGTTCGGCCCAATGAAGAA	R: CCCTCATGGACATGTTTGCA
TFAP2C	F: TCCCCACCTGAATGCTTAAATG	R: GAGAGTCACATGAGCGGCTTT
BCL2L11	F: ACAAGGTAATCCTGAAGGCAATCA	R: GACAGCAGGGAGGATCTTCTCA
GATA6	F: TTCCATTCCCATGACTCCAACT	R: AGCTCGCTGTTCTCGGGATT
EBF2	F: CGCACGGAACAGGACCTCTA	R: ACAGCTTTTCTTTTCGCAGCAT
HOXA3	F: GAATCGCCGCATGAAGTACA	R: TCATACGGGACGCTGTTGAC
CADM2	F: CATCGACTCCTTTTCCACAAGAA	R: ACAGCTTTTCTTTTCGCAGCAT

### Cell proliferation and colony formation assay

Transfected SCC-25 cells were seeded into 96-well plates (2 × 10^3^ cells per well) for 24, 48, and 72 h measured using CCK-8 assay (Solarbio, China) for cell proliferation assay. We measured the absorbance at 450 nm of the cells to determine their viability (Thermo Fisher Scientific, USA). Colony formation assays were performed using cells transplanted into six-well plates at a rate of one per well for a period of two weeks before 0.1% Crystal Violet was used to stain for viable colonies.

### Apoptosis assay

The cells were harvested, which were transfected and clutured in six-well plates (1 × 10^6^ cells per well) for 48 h, and stained with recombinant annexin V-FITC (Fluorescein isothiocyanate-labeled) for 10 min at room temperature. After that, cells were stained for 10 min at room temperature with Propidium Iodide (PI). Flow cytometry (Apogee, UK) was performed immediately after staining.

### Wound healing and transwell assays

Transfected SCC-25 cells were incubated until reaching to 100% confluence. Cells were washed three times with phosphate-buffered saline (PBS) after scratching the linear wound with a 200 mm pipette tip. Image of wound were taken with microscope at 0 and 24 h respectively. In migration assay, transfected SCC-25 cells, with 24 h starvation, were collected and seeded into the upper champer with serum-free medium. Filling the lower chamber with complete medium. In cell invasion assays, Cell invasion abilities were assessed in chambers coated with Matrigel. For 15 min, 0.1% crystal violet was applied to the cells after they had been incubated for 24 h. The migrated cells were observed with an optical microscope (Nikon, Japan) in five randomly selected areas.

### Western blot analysis

The total protein was extracted using the kit (Beyotime Biotechnology, China), which included five samples of OSCC tissue paired with adjacent normal tissues. SDS-containing polyacrylamide gels were used to separate protein samples for transfer to polyvinylidene fluoride membranes (Millipore, USA). The membrane was treated with the primary antibody for an overnight period at 4 °C after being blocked with 5 percent BSA at room temperature. The primary antibodies were anti-p62 (1:1000; Proteintech, cat.no. 18420-1-AP), anti-LC3 (1:1000; Proteintech, cat.no. 14600-1-AP), anti-ATG5 (1:1000; Proteintech, cat.no. 10181-2-AP), anti-Bim (1:1000; Cell Signaling Technology, cat.no. #2933), anti-BCL-2 (1:1000; Proteintech, cat.no. 12789-1-AP), anti-Bax (1:1000; Cell Signaling Technology, cat.no. #14796), anti-GAPDH (1:2000; Elabscience, cat. no. E-AB-20072), and anti-Beclin-1 (1:1000; Proteintech, cat.no. 11306-1-AP). After three washes with TBST, the membrane was incubated for 1 h at room temperature with goat anti-rabbit IgG (1:10,000; Proteintech, cat.no. 10285-1-AP).

### IHC staining

IHC was performed on paraffin-embedded tissues. It was conducted with rabbit anti-LC3 (1:200; Proteintech, cat.no. 14600-1-AP), rabbit anti-Bim (1:200; Proteintech, cat.no. 22037-1-AP), or rabbit anti-p62 (1:200; Proteintech, cat.no. 18420-1-AP) as the primary antibody. Antigen was removed at 95 °C for 20 min with 10 mM sodium citrate (pH 6.0). After blockading the sections with endogenous peroxidases and goat serum for 10 min, the primary antibodies were incubated overnight at 4 °C. DAB (Sangon Biotech, China) was used to observe tissue antigen. Finally, hematoxylin counterstained the specimens.

### Immunofluorescent staining

For 12 h, SCC-25 cells were cultured in 24-well plates with coverslips in DMEM containing 10% FBS. After the treatment, the medium was removed and PBS were used to wash the coverslips with cells. After cells were fixed in 4% paraformaldehyde, they were permeable for 10 min in 0.1% Triton X-100 at room temperature. The cells were blocked by 5% bull serum albumin and incubated with primary antibody anti-LC3 (Proteintech, cat.no. 14600-1-AP) overnight at 4 °C. on the second day, the Anti-Rabbit econdary antibody (1:100; Proteintech, cat.no. SA00007-2) was incubated the cells for 1 h and the cells nucleus were stained by DAPI (Solarbio, China) for 5 min. Images were taken by laser scanning confocal microscope.

### Autophagosome detection by stubRFP‐sensGFP‐LC3

According to the manufacturer’s instruction, SCC-25 cells were transfected with stubRFP‐sensGFP‐LC3 Adenovirus (Genechem, China) and seeded in 15 mm glass‐bottom culture dishes. Cells were observed and recorded under a laser scanning confocal microscope (Leica, Germany).

### Transmission electron microscopy

The pre-treated cells were harvested and fixed in the Glutaraldehyde, 2.5% (Solarbio). The next steps were accomplished by the experts and the images were observed with a transmission electron microscope (JEM-1200, Jeol, Japan).

### microRNA target gene prediction

The potential target genes of miR-10b were determined using Target Scan (www.targetscan.org) [26], miRDB (http://mirdb.org/) [27], microT_CDS (http://diana.imis.athena-innovation.gr/DianaTools/index.php?r=microT_CDS/index) [28], EIMMo (https://mirz.unibas.ch/ElMMo3/index.php) [29]. As a further measure to reduce the number of false target genes, top 50 target genes of each data set were also considered as potential targets.

### Luciferase reporter assay

The mutated (MUT) 3′-UTR or wild-type (WT) 3′-UTR of Bim was designed and cloned to EGFP plasmids. MiR-10b mimics or negative control along with WT or MUT 3′-UTR plasmids were co-transfected into SCC-25 cells. The luciferase activity was analyzed 48 h after transfection using a dual-Luciferase reporter Assay System (Promega, USA).

### Xenotransplantation

The Affiliated Hospital of Qingdao University’s Animal Care and Use Committee approved all animal experiments, which were conducted in accordance with institutional guidelines. In this experiment, two groups of female BALB/c nude mice were used supplied by Sino-British Sipper/BK Lab Animal Ltd(Shanghai, China). All laboratory animals were maintained in an environment free of specific pathogens at the Department of Laboratory Animals. A subcutaneous injection of 100 ml PBS containing either lv-ANTI-miR-10b (GeneChem, China), or control SCC-25 cells (5 × 10^6^) was then performed in mice. Every seven days, the tumor volume was measured, V = *a* × *b*^2^/2 was used to calculated the tumor volume. The tumors were collected and weighed after all nude mice were dislocated after 28 days.

### Statistical analysis

One-way analysis of variance (ANOVA), independent t-test, and paired t-test were used in all statistical analyses in the SPSS 22.0 software (IBM, Armonk, NY, USA). Data were indicated as mean ± SD. *p < 0.05, **p < 0.01 were considered Statistical significance.

## Supplementary information

The full length uncropped original western blots

## Data Availability

The datasets generated and/or analyzed during the current study are available from the corresponding authors on reasonable request.
