# Supplementary material for: The microRNA-10b-Bim axis promotes cancer progression through activating autophagy in oral squamous cell carcinoma
Source: Cell Death Discov. 2022 Aug 25;8:373. doi: 10.1038/s41420-022-01168-1 (PMC9411559; doi:10.1038/s41420-022-01168-1)

Fig 2 SCC25 control miR-10b inhibitor miR-10b mimics

p62


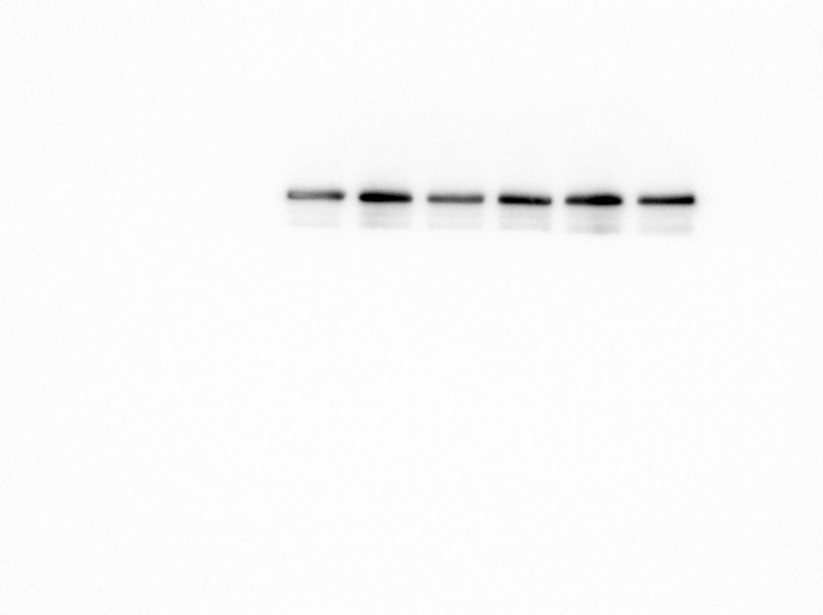


LC3


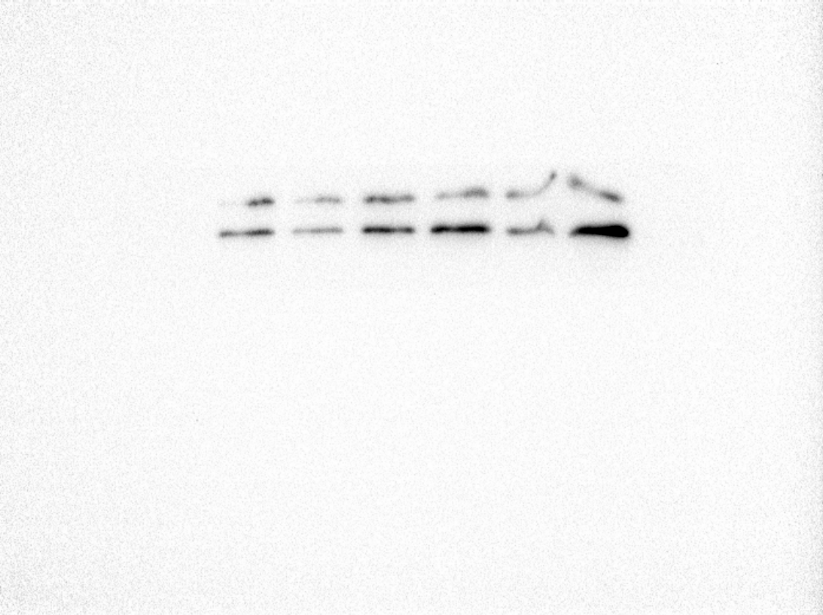


GAPDH


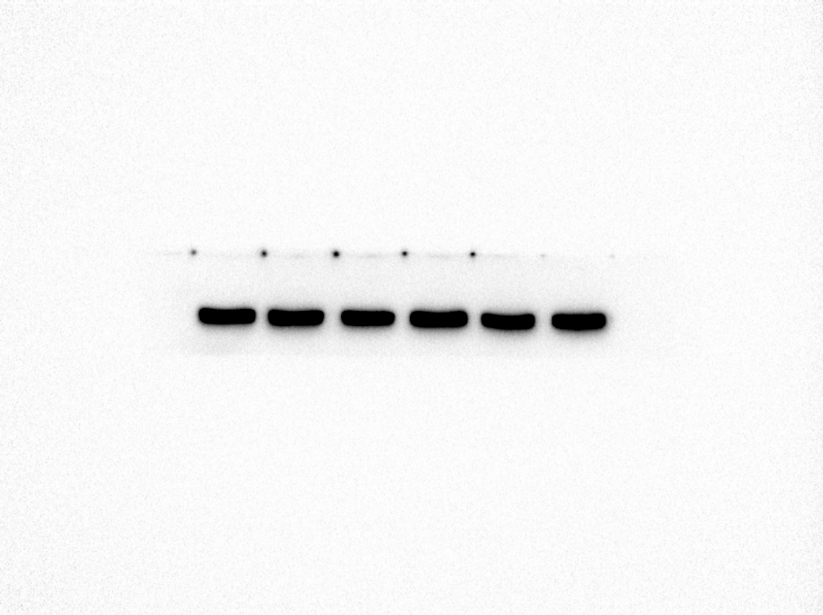


Fig3 SCC25 miR-10b mimics miR-10b mimics+siATG5

ATG5


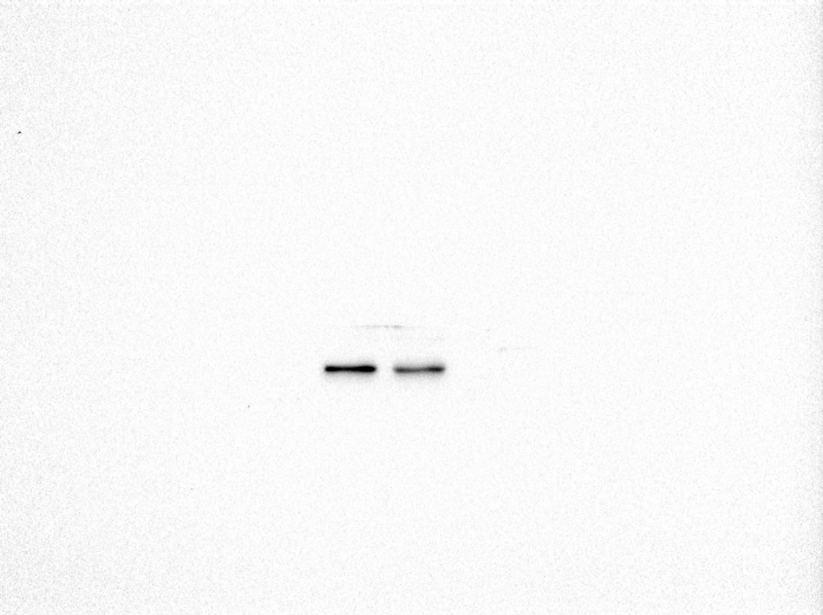


GAPDH


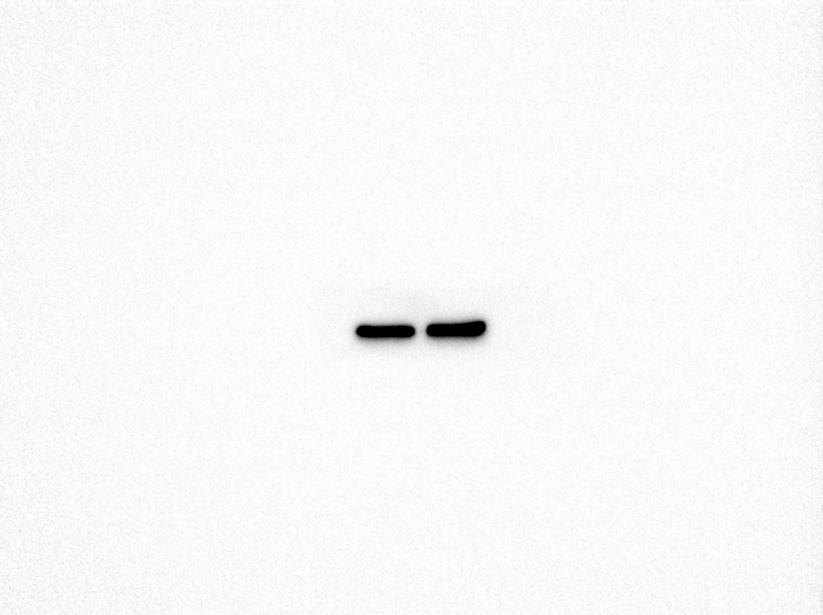


Fig4 SCC25 Bim

Bim


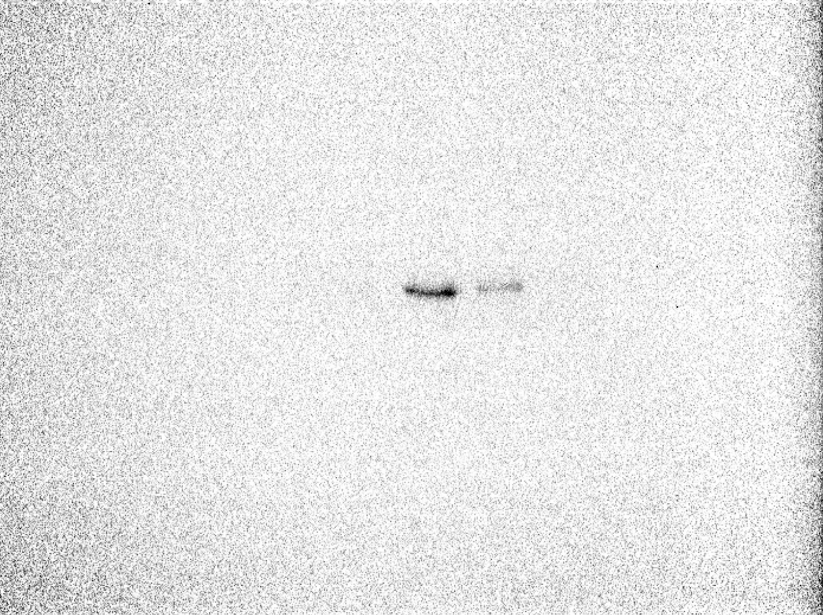


GAPDH


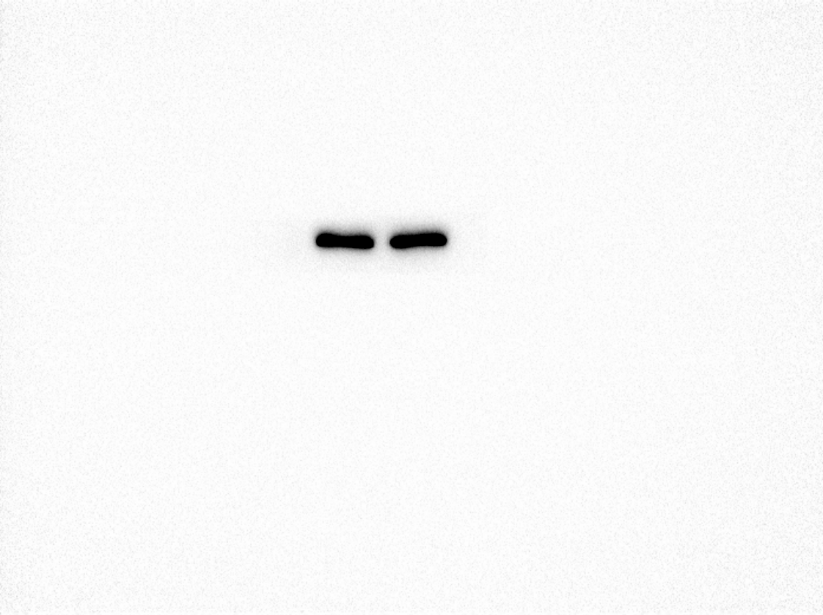


Fig6 SCC25 Control Bim Bim+miR-10b mimics

LC3


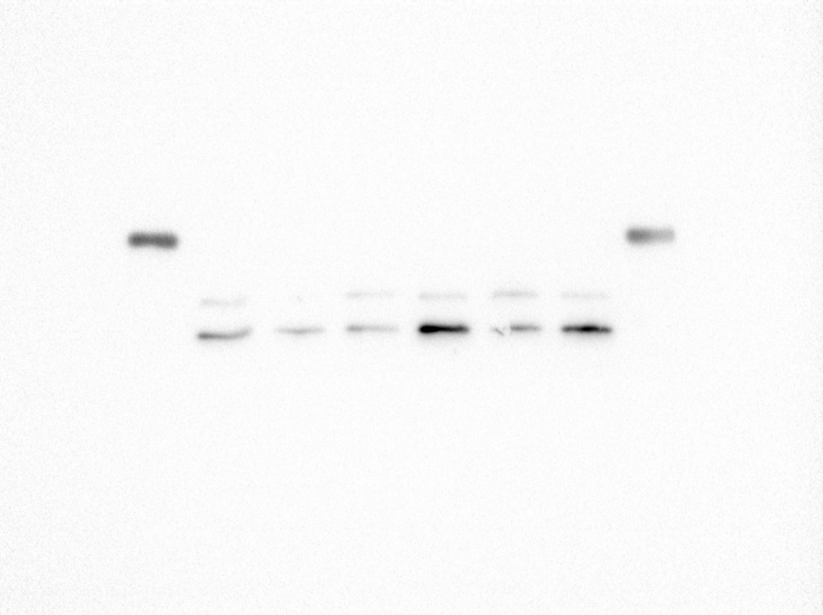


GAPDH


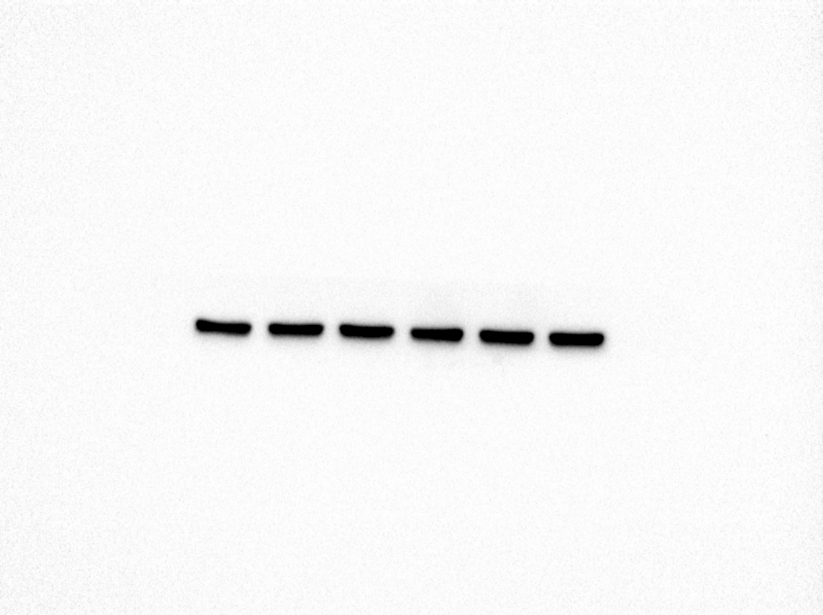


P62


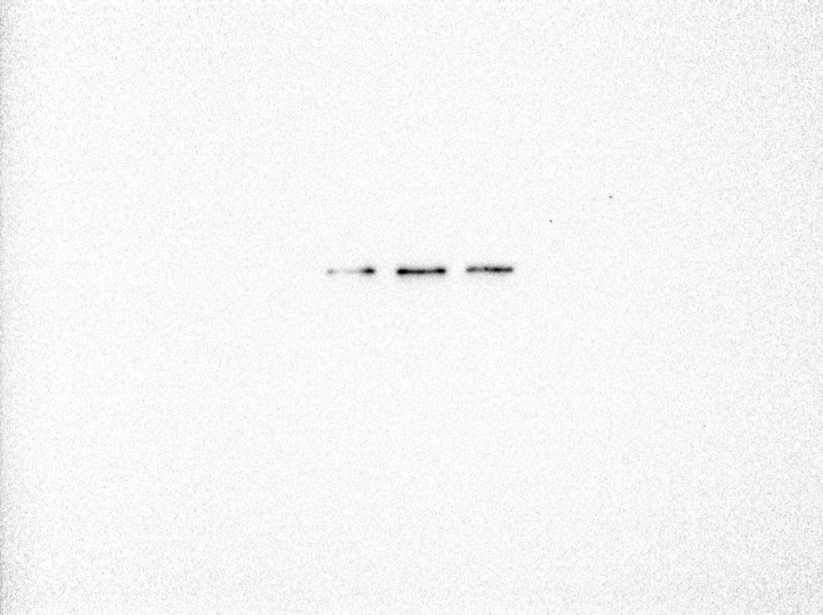


Beclin1


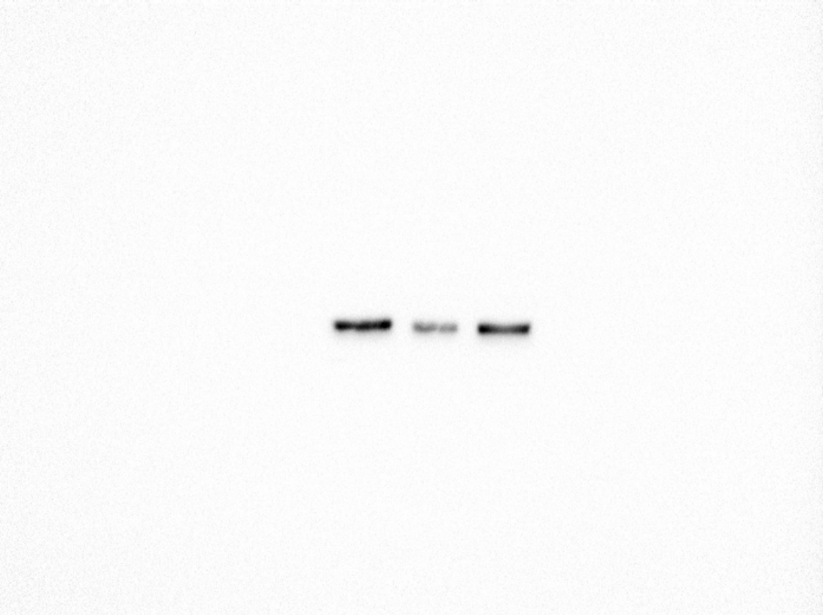


Bim


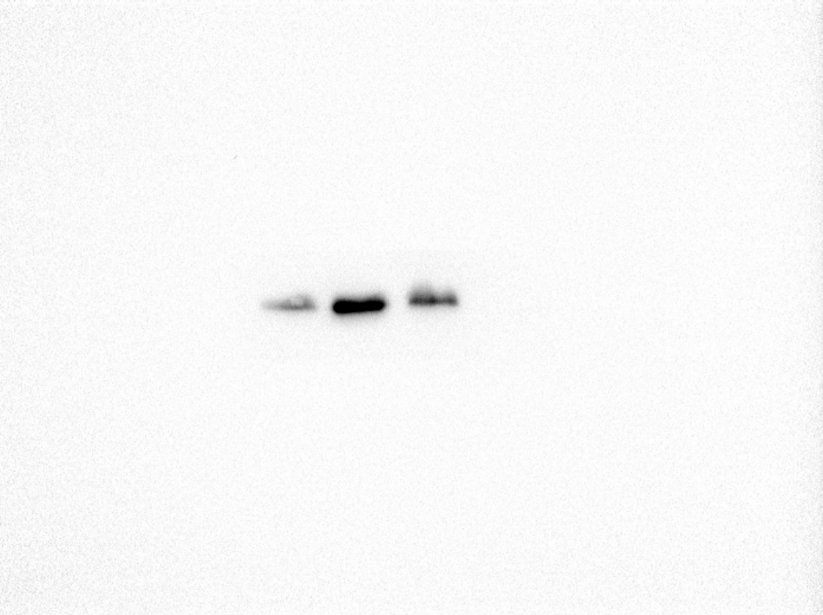


GAPDH


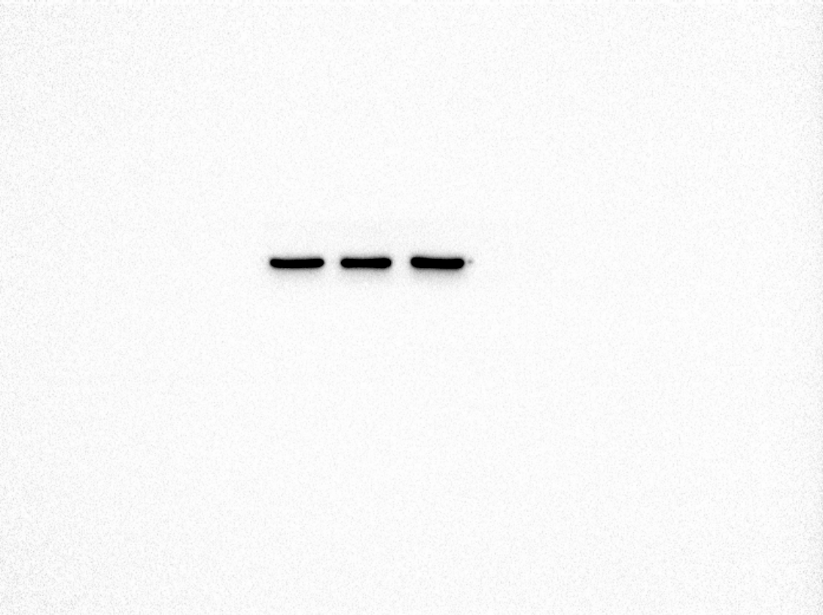

Supplement: Supplementary file 1 — The full length uncropped original western blots [file 41420_2022_1168_MOESM1_ESM.docx]
